# Ferroptosis: The dawn of reversing drug resistance in digestive cancers

**DOI:** 10.1016/j.gendis.2025.101873

**Published:** 2025-09-26

**Authors:** Wenjie Chen, Liang Han, Jizhou Wang, Linjiang Song

**Affiliations:** a School of Medical and Life Sciences, Chengdu University of Traditional Chinese Medicine, Chengdu, Sichuan 611137, China; b Hospital of Chengdu University of Traditional Chinese Medicine, School of Clinical Medicine, Chengdu University of Traditional Chinese Medicine, Chengdu, Sichuan 610075, China

**Keywords:** Chemotherapy, Digestive cancers, Drug resistance, Ferroptosis, Targeted therapy

## Abstract

Ferroptosis, a form of iron-dependent cell death characterized by lipid peroxidation, has emerged as a promising strategy to overcome resistance to chemotherapy. This review explores the mechanisms of ferroptosis and its potential to reverse drug resistance in digestive system cancers. We summarize recent advances in understanding the GPX4-regulated pathway, iron metabolism, and lipid peroxidation as key drivers of ferroptosis. It also highlights the roles of tumor heterogeneity, tumor–stroma interactions, abnormal apoptosis, metabolic alterations, and the tumor microenvironment in drug resistance. Specific mechanisms of ferroptosis resistance in esophageal squamous cell carcinoma, gastric cancer, hepatocellular carcinoma, pancreatic ductal adenocarcinoma, and colorectal cancer are discussed, along with strategies to induce ferroptosis to reverse drug resistance. Future research should focus on translating these findings into clinical applications through targeted therapies and combination treatments to improve patient outcomes.

## Introduction

Cancers of the digestive system include esophageal squamous cell carcinoma, gastric cancer, colorectal cancer, hepatocellular carcinoma, pancreatic cancer, and others that develop in the digestive tract from the mouth to the anus and related organs.^1^ Cancers of the digestive system account for one-quarter of malignancies recorded globally and more than one-third of all cancer-related fatalities.^2^ Digestive system cancers are among the most prevalent malignancies, which pose a significant threat to human health.^3^

At the same time, current diagnostic and treatment technologies have limitations, including low patient cure rates and severe side effects. Although chemotherapy is widely used to treat cancers, one key factor contributing to chemotherapy failure is cancer cell resistance to anti-cancer drugs.^4^ As a result, it is crucial to develop innovative approaches to treating cancers of the digestive system. Drug resistance is often described as the lower sensitivity or ineffectiveness of a pathogen or cancer cells to a drug after multiple exposures to the drug, which can be categorized as either primary or acquired, depending on the underlying causes.^5^ Extensive research has been conducted on pharmacological therapies to combat cancer, with notable therapeutic success in select patients. However, the persistence of resistance to drugs creates a significant barrier to achieving effective therapy results.^6^ Hence, a profound exploration into solving drug resistance has become imperative. Ferroptosis sheds new light on this regard.

Ferroptosis, which is triggered by intracellular iron, is a novel type of controlled cell death distinct from apoptosis, necrosis, and autophagy. In 2012, the term “ferroptosis” was first introduced to describe iron-dependent cell death resulting from the accumulation of reactive oxygen species in lipids.^7^ Due to its unique metabolic features, it can be utilized to examine a wide range of disorders, including malignant tumors.^8^ The most common hallmarks of ferroptosis are abnormal iron metabolism and lipid peroxidation.^8^ Ferroptosis is influenced by iron, lipid, and amino acid metabolism, as well as the GPX4 (glutathione peroxidase 4) pathway. Numerous inducers and inhibitors have also been found. Numerous studies have demonstrated that ferroptosis is of great significance in cancer suppression, which paves the way for novel cancer treatment approaches. The development of resistance to cancer therapy remains a significant concern.^9^ A variety of clinical studies have attempted to overcome drug resistance. Interestingly, there is a notable link between ferroptosis and drug resistance. Accumulating evidence, including the results presented herein, highlights resistance to ferroptosis as a bona fide and clinically relevant mechanism of tumor drug resistance, and it has been demonstrated that inducing ferroptosis can reverse the occurrence of drug resistance.

## Mechanisms of ferroptosis

**Figure 1:**
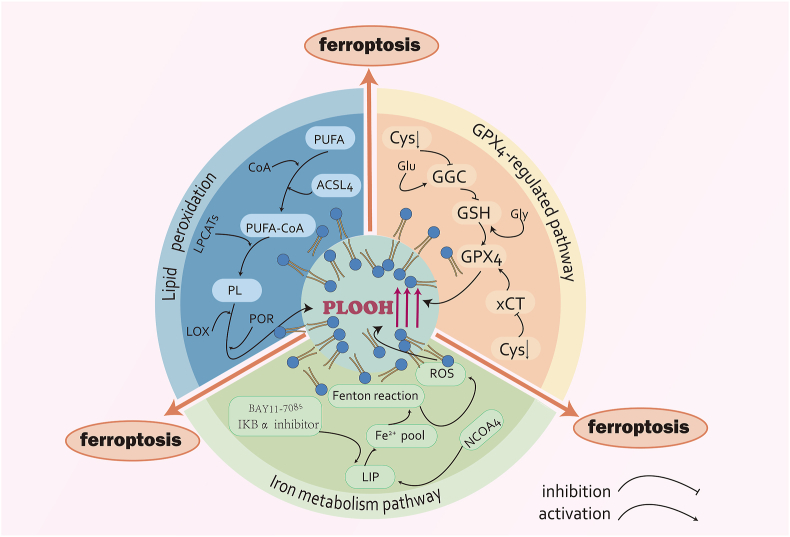
The mechanisms of ferroptosis. ①GPX4-regulated pathway: GSH is essential for GPX4 to perform its normal physiological function. The inhibition of GSH synthesis through the inhibition or reduction of cysteine can also indirectly inhibit GPX4. This occurs by depriving cells of cysteine and inhibiting the xCT system, two pathways that ultimately lead to cell membrane damage and ferroptosis due to PLOOH accumulation caused by GPX4 inactivation. ②Iron metabolism pathway: Intracellular LIP is enhanced by the overexpression of the IκBα inhibitor BAY 11–7085 and nuclear receptor coactivator 4, which promotes ferritin disintegration. This, in turn, generates PLOOH and free radicals via the Fenton reaction. Ultimately, ferroptosis is the result of lipid peroxidation, which is induced by the production of ROS. ③Lipid peroxidation: Catalyzed by ACSL4, PUFA forms PUFA-CoA with CoA and generates PL via LPCAT, which produces large amounts of PLOOH in response to LOX and cytochrome P450 oxidoreductase (POR) and ultimately induces ferroptosis.

Research on the mechanisms underlying ferroptosis has advanced rapidly in the past few years. According to recent studies, the GPX4-regulated pathway, lipid peroxidation, and iron metabolism are the main characteristics of ferroptosis (Fig. 1). These studies have provided a strong theoretical foundation for the initiation of ferroptosis.^9^

### GPX4-regulated pathway

A recent investigation using a range of ferroptosis-inducing agents (FINs) revealed that FINs significantly decreased GPX4 levels, either directly or indirectly through glutathione (GSH) depletion. Thus, they concluded that GPX4 is the primary regulator of ferroptosis.^10^ GPX4 appeared to be essential for catalyzing the reduction of phospholipid hydroperoxides (PLOOH) into corresponding phospholipid alcohols because it was a glutathione peroxidase in mammals.^11^ GSH was necessary for GPX4 to function physiologically as expected.^12^ The enzyme glutamate-cysteine ligase catalyzed the synthesis of intracellular GSH. For this reason, the rate-limiting amino acid for the synthesis of GSH was cysteine, which was absorbed as cystine through the cystine/glutamate antiporter (xCT) system.^13^ Therefore, inhibiting the xCT system to prevent cells from receiving cysteine also helped. Hence, depleting cysteine from cells led to indirect inhibition of GPX4 via the suppression of the xCT system. Thus, PLOOH accumulation resulting from GPX4 inactivation may lead to damage to cell membranes and ferroptosis cell death. Redox-regulated cell death and neurodegeneration were demonstrated to result from GPX4 inactivation in 2008, both *in vivo* and *ex vivo*.^14^ Research has indicated that genetic inhibition of GPX4 can induce ferroptosis in cancer cells and inhibit cancer growth *in vivo*.^10^ As a crucial regulator of ferroptosis, GPX4 is governed by the expression of non-coding RNAs. A study found that miR-324-3p inhibits GPX4 and induces ferroptosis, reversing cisplatin resistance in NSCLC cells.^15^ These results suggest a vital role for the GPX4-regulated pathway in cancer biology.

### Iron metabolism pathway

Ferroptosis is a form of cellular mortality that is dependent on iron and is distinguished by an increase in the labile iron pool, also known as the minor Fe^2+^ pool. In 1997, it was determined that the primary mechanisms for cellular iron absorption were transferrin endocytosis and the binding of serum transferrin to the transferrin receptor.^16^ According to a study in 2016, autophagy broke down ferritin in fibroblasts and cancer cells, which led to ferroptosis. The level of intracellular LIP was increased by the overexpression of nuclear receptor coactivator 4, which promotes ferritin disintegration.^17^ An increase in the intracellular LIP may contribute to phospholipid peroxidation, which in turn generates PLOOH and free radicals through the Fenton reaction.^18^ Additionally, the majority of reactive oxygen species (ROS) production in cells was catalyzed by iron. Ultimately, ferroptosis was the result of lipid peroxidation, which was induced by the production of ROS.^19^ It has been demonstrated that cancer cells require a greater amount of iron to persist than normal cells.^20^ Ferroptosis induction may be a valuable target for cancer treatment, as it increases intracellular iron levels and facilitates iron assimilation in rapidly developing cancer cells. Research has discovered that the growth of lung cancer cells *in vivo* is inhibited and that lung cancer cells are more susceptible to ferroptosis when NFS1 is reduced to increase the level of intracellular LIP.^21^ Furthermore, Chang et al discovered that the well-known IκBα inhibitor BAY 11–7085 improved cancer cell ferroptosis by increasing the LIP, which activated heme oxygenase-1.^22^ When coupled, the iron metabolism route is modulated to induce ferroptosis in cancer cells therapeutically.

### Lipid peroxidation

Given that increased lipid peroxidation is the defining feature of ferroptosis, it is firmly believed that lipid peroxide metabolism is crucial for the process. Ferroptosis is most likely facilitated by the peroxidation of membrane phospholipids to produce PLOOH, which is subsequently decomposed to produce malondialdehyde or 4-hydroxynonenal molecules. Lipid peroxidation end products led to the permeabilization and instability of cell membranes, ultimately causing cell death.^23^ The enzyme acyl-CoA synthetase long-chain family member 4 (ACSL4) promotes the binding of polyunsaturated fatty acids (PUFAs) to coenzyme A (CoA) during nonenzymatic lipid peroxidation, leading to the synthesis of acyl-CoA. A range of lysophosphatidylcholine acyltransferases (LPCATs) can re-esterify acyl-CoA in phospholipids to produce phospholipids. The sensitivity to ferroptosis can be consequently assessed by adjusting the expression of LPCATs and ACSL4.^24^^,^^25^ Lipoxygenases (LOXs) and cytochrome P450 oxidoreductase (PORs) are active enzymes that can induce the production of PLOOH during enzymatic lipid peroxidation. Lipid-oxidase enzymes (LOXs), which lack heme iron, directly facilitated the deoxygenation of esterified and freed PUFAs to generate PLOOH.^26^ Previous work has demonstrated that the up-regulation of LOX-5, LOX-12, and LOX-15 is associated with increased vulnerability of cells to ferroptosis. Furthermore, studies have shown that LOX inhibitors are potent antioxidants that protected cells against lipid peroxidation.^27^ In 2020, Zou et al demonstrated that POR was essential for ferroptosis cell death in cancer cells through genome-wide CRISPR-Cas9-mediated suppressor screening.^28^ P450 has been shown in prior studies to induce the peroxidation of PUFAs by absorbing electrons from POR.^29^ Furthermore, genetic depletion has been employed in several lineages and cell states to demonstrate the pro-ferroptotic role of POR.^30^

### Crosstalk among GPX4, iron metabolism, and lipid peroxidation in ferroptosis

Ferroptosis is executed through an intricate interplay of iron-dependent lipid peroxidation, GPX4 activity, and metabolic rewiring of iron and lipids. This crosstalk manifests distinctly in cancer, where dysregulated signaling amplifies vulnerability or resistance to ferroptosis death.^31^ Iron acts as a catalytic linchpin, wherein labile Fe^2+^ drives Fenton reactions that convert PLOOH into reactive alkoxyl radicals, propagating peroxidation chains. This process is fueled by iron import via the transferrin receptor or ferritinophagy-mediated iron release, both of which are up-regulated in malignancies, such as *p53*-mutant tumors. Crucially, the susceptibility of membranes to peroxidation is governed by lipid metabolism. Enzymes such as ACSL4 and LPCAT3 incorporate pro-ferroptotic polyunsaturated fatty acids into phospholipids, while SCD1(stearoyl-coenzyme desaturase 1) promotes the synthesis of monounsaturated fatty acids that competitively inhibit lipid peroxidation. GPX4 serves as the primary defense agent by reducing PLOOH to inert alcohols, but its activity is compromised by system Xc^−^ inhibition or direct inactivation.^32^

In cancers, oncogenic pathways rewire this triad to modulate ferroptosis sensitivity. For instance, in VHL-mutant clear-cell renal cell carcinoma, HIF-2α up-regulates the lipid droplet protein HILPDA, liberating PUFAs for peroxidation while simultaneously suppressing SCD1 to reduce protective MUFA-PLs. Conversely, hyperactivation of PI3K-AKT-mTOR signaling in breast or prostate cancer induces SREBP1-dependent SCD1 transcription, enriching membranes with MUFA-PLs to resist peroxidation, mTORC1 also enhances GPX4 translation through Sec-tRNA modification, thereby creating a dual resistance mechanism. Paradoxically, tumors with KEAP1 or LKB1 mutations become addicted to SCD1, rendering them sensitive to GPX4 inhibition.^10^^,^^33^

## Mechanisms of tumor drug resistance

Tumor resistance mechanisms are numerous and complicated. Tumors are defined by genetic diversity and selective evolution, which results in drug resistance. Genetic differences are typically the reason. As a result, whether primary or acquired, drug resistance is a separate and irreversible phenomenon.^34^, ^35^, ^36^ Genetic alterations that confer treatment resistance to the specific targeted inhibitors may already exist in tumor cells.^37^

Primary resistance occurs when there is no tumor shrinkage or remission following first-line therapy. It is typically triggered by a genetic mutation, aberrant behavior of tumor cells, or a rapid response of tumor cells to treatment.^38^^,^^39^

Acquired resistance is more common than primary resistance. The term relates to the resistance that develops throughout therapy. The two primary hypotheses of acquired resistance today are pre-existing and evolutionary. Prior studies have shown that tumors exhibit several types of clonal heterogeneity and that rare subclones may already be drug resistant before treatment starts. After receiving medication, these resistant subclones kept proliferating, which leads to drug-induced tumor recurrence.^40^^,^^41^

**Figure 2:**
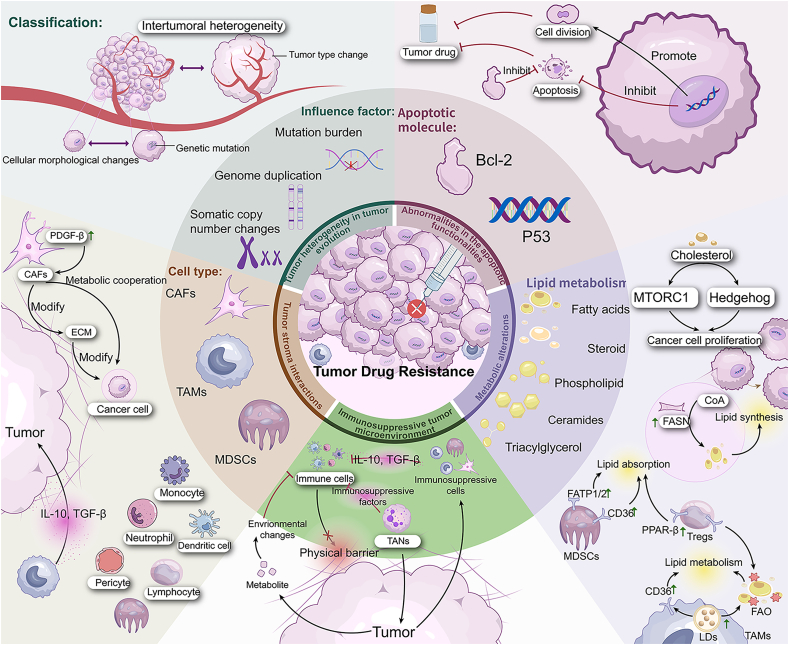
The mechanisms of tumor drug resistance. ①Role of tumor heterogeneity: Tumor heterogeneity is classified into two types: intratumoral heterogeneity and intertumoral heterogeneity. Mutational burden, somatic copy number changes, and genome doubling influence tumor heterogeneity and development. ②Abnormalities in apoptotic functionalities: The mutant P53 gene promotes cell division and inhibits apoptosis. When Bcl-2 is overexpressed, the intracellular drugs that cause damage fail to induce effective death signals, considerably slowing tumor cell death and delaying the establishment of drug resistance.③Tumor–stroma interactions: CAFs promote their proliferation by overexpressing PDGF receptor-β. CAFs aggressively interact with cancer cells through metabolic cooperation, improving their ability to penetrate and survive while modifying the extracellular matrix and promoting tumor cell growth and proliferation. Immune cells in the immunosuppressive tumor microenvironment (TME) are also involved. ④Metabolic alterations: Lipid metabolism is used by cancer cells as a means of overcoming various obstacles encountered during the metastatic cascade. Intratumoral Tregs are immunomodulators that activate the transcription factors CD36 and PPAR-b, which boost FAO and fat absorption. These cells can up-regulate the SREBP transcriptional program to boost FASN-dependent lipid production and PD-1-mediated inhibitory receptor signaling. Their ability to suppress the immune system gradually increases, which encourages the formation of tumors. ⑤Immunosuppressive tumor microenvironment (TME): An immunosuppressive tumor microenvironment develops as cancer spreads, changing the stromal and immune cells in the area to make it more difficult for the immune system to recognize, infiltrate, and function. This includes the recruitment of immune-suppressive cell types, changes in the local metabolic environment, physical barriers to immune cell infiltration, and an increase in soluble substances.

The mechanisms of tumor drug resistance were sorted out in Figure 2.

### Tumor heterogeneity in tumor evolution

Tumors from the same species may differ in features because internal tumor cells have various genotypes. Tumor heterogeneity refers to this phenomenon. The most common factor that promotes tumor resistance is tumor heterogeneity.^42^, ^43^, ^44^

Tumor heterogeneity is classified into two types: intratumoral heterogeneity, which refers to the presence of multiple subclones within a single tumor with distinct molecular profiles,^45^ and intertumoral heterogeneity, which denotes the molecular differences between tumors from different sites within the same patient. Intratumoral heterogeneity exists in two forms: temporal and spatial heterogeneity.

Tumor heterogeneity and development are influenced by mutational burden, somatic copy number changes, and genome doubling. Low intratumoral heterogeneity was observed in Caucasian non-small cell lung cancer patients whose multi-region sequencing studies revealed a substantial smoking-associated mutational load for clonal mutations.^45^

### Tumor stroma interactions

A considerable amount of extracellular matrix (ECM) and a variety of support cells, such as pericytes, endothelial cells, cancer-associated fibroblasts (CAFs), and immunologic cells,^46^ which include the most common cell types (lymphocytes, neutrophils, dendritic cells, and monocytes), are found in the nonneoplastic portion of the tumor microenvironment (TME), also referred to as the tumor stroma. Mesenchymal stromal cells (MSCs),^47^ platelets,^48^^,^^49^ and myeloid-derived suppressor cells (MDSCs) are other, less often used components. Stromal cells interact with tumor cells and the ECM through the release of chemokines such as growth factors, enzymes, extracellular vesicles, and miRNAs. These interactions alter cancer metabolic pathways and regulate gene and protein expression.^50^ Different cell types can either encourage or hinder the growth of tumors, depending on the cellular environment.^51^ The diverse spectrum of immune and inflammatory cells that make up this stromal mass^52^ creates a physical barrier that increases chemoresistance and prevents chemotherapeutic medications from reaching cancerous cells.

The ECM has a role in paracrine cellular communication in addition to supporting and structuring individual cells inside tissues and organs.^53^ Proteoglycans, glycosaminoglycans, fibrous molecules, proteins, and other highly organized macromolecules make up the extracellular matrix. Currently, approximately 300 proteins have been identified as components of the extracellular matrix.^54^ Each organ's distinct ECM compositions satisfy its unique needs and functions throughout embryonic development.^55^

Stromal cells, typically located around solid tumors, influence carcinogenesis and even resistance to anticancer treatments via interactions with cancer cells and with one another. As one of the most common cell types in the tumor stroma, CAFs are crucial to the development of cancer. α-SMA-positive cells are frequently used to identify CAFs. CAFs promote their proliferation by overexpressing the platelet-derived growth factor (PDGF) receptor-β and do not revert to their inactivated state, in contrast to noncancerous myofibroblasts.^56^ CAFs aggressively interact with cancer cells through metabolic cooperation, improving their ability to penetrate and survive while also modifying the extracellular matrix and promoting tumor cell growth and proliferation.^57^^,^^58^ For instance, before exporting pyruvate, which cancer cells utilize for metabolism, CAFs collect and break down extracellular lactate.^50^ Due to the pro-carcinogenic action of TGF-β, CAFs increase the density of collagen in tumor tissues, resulting in increased cell proliferation, increased matrix stiffness, and hypoxia.^56^

Tumor-associated macrophages (TAMs), lymphocytes, natural killer (NK) cells, and DCs are among the numerous immune cells present in the tumor stroma that are required for tumor management. Immunosuppressive mechanisms include M2-type TAMs, MDSCs, and regulatory T cells. On the other hand, after cancer cells are recognized, CD8^+^ T lymphocytes are responsible for eliminating their surface antigens. However, interactions with TAMs, CAFs, and cancer cells inhibit, degrade, or prevent these T cells from reaching the tumor parenchyma, thereby promoting the growth of cancer cells.^59^ Cancer cells control the process of TAM differentiation, which attracts monocytes to the TME.^60^ TAMs, in turn, promote angiogenesis and ECM remodeling.^61^ TAMs limit adaptive immunity by releasing anti-inflammatory cytokines such as IL-10, CCL22, and TGF-β, making them promising targets for immunotherapy.^47^

### Immunosuppressive tumor microenvironment

As cancer progresses, an immunosuppressive TME is formed, altering local stromal and immune cells to impede immune detection, infiltration, and function. This includes physical barriers to immune cell infiltration, alterations in the local metabolic environment, recruitment of immune-suppressive cell types, and an increase in soluble factors.^62^ Fibroblasts, the most common type of stromal cell in the tumor TME, control the infiltration and activation of cancer-fighting immune cells, which is essential for changing the TME.^63^ Moreover, they facilitate the development of drug resistance, metastasis, and tumor cell proliferation through various mechanisms. By creating an environment that is favorable to cancer cells, CAFs, a subtype of fibroblasts that promote tumor growth, act as “soil” for cancer “seeds”. It is common to equate tumors to “unhealable wounds”, and when tissue damage appears, CAFs try to repair it, which leads to a substantial tumor accumulation.^64^

A considerable proportion of the immune cells within the TME are neutrophils, and their increased infiltration is associated with a less favorable prognosis in the majority of solid tumors.^65^^,^^66^ They exhibit functional plasticity and are impacted by their surroundings in their diverse roles in the genesis of solid tumors.^66^ Some neutrophils are capable of eliminating cancer cells through the generation of ROS and the release of neutrophil elastase,^67^^,^^68^ the majority of tumor-associated neutrophils (TANs) are polarized by TME mediators and promote tumor development.^69^ By encouraging the growth of cancer cells, angiogenesis, and tumor dispersion through the generation of protumor chemicals, TANs improve the development of tumors.^70^ There is a unique tumor mechanism in TANs. When the TME is active, polarized neutrophils form an immunosuppressive TME by producing CCL5, a chemokine ligand, and Nectin 2, which has been identified. Neutrophils have variable functional variation based on signals specific to different tissues, age, and maturity.^71^ They can have a variety of effects on tumor growth as well as anti- and protumor activity due to their capacity to change their functional phenotypes in response to diverse situations.

### Abnormalities in the apoptotic functionalities

Apoptosis, another name for programmed cell death, is a mechanism that has evolved to be preserved and is necessary for tissue homeostasis and organism growth.^72^ However, in pathological conditions such as cancer, cells become incapable of dying through apoptosis, which leads to unchecked cell division. A significant number of proteins that are necessary to stop the apoptotic cascade from starting are typically overexpressed in cancer cells. There are several ways for cells to avoid being killed by a program, one of which is by making anti-apoptotic compounds. Recent research has suggested that apoptosis resistance is a complex phenomenon involving several supporting factors that either cooperate with or operate independently of Bcl-2 signaling.^73^

Apoptotic dysfunction has a significant impact on the development and spread of tumors. Furthermore, they induce multidrug resistance (MDR) in tumor cells, rendering them more susceptible to chemotherapeutic agents that induce apoptosis.^74^ For example, the wild-type P53 gene supports healthy cell division, triggers programmed cell death, and stops cancer from spreading. However, a mutant p53 gene results in irreversible damage to DNA, which in turn leads to the formation of genetically unstable cells that ultimately transform into cancer.^75^^,^^76^

Additionally, the Bcl-2 gene is considered one of the key oncogenes in the field of apoptosis studies. When Bcl-2 is overexpressed, the intracellular drugs that cause damage fail to induce effective death signals, thereby considerably slowing tumor cell death and delaying the establishment of drug resistance.^76^ One reason might be that increased Bcl-2 protein synthesis in cells resulted in a more substantial anti-apoptotic effect, which reduces the quantity of apoptosis caused by chemotherapeutic drugs and decreases cell sensitivity to such treatments. Additionally, Bcl-2 overexpression enhanced cell resistance to chemotherapeutic agents such as cisplatin, methotrexate, and docetaxel.^77^

### Metabolic alterations

Metastasis is mainly caused by metabolic alterations. Although glucose is most likely the primary metabolic substrate of rapidly developing tumors, lipids, amino acids (such as glutamine), and glycolytic metabolites (such as pyruvate and lactate) may also speed up the metastatic process.^78^ A growing body of evidence suggests that lipid metabolism often improves at various stages of cancer progression. These modifications provide tumor cells with energy, but they also change membrane composition, signaling, and epigenetics, which encourages metastasis.^78^ Moreover, the metabolic connections between the surrounding cells and the tumor are encouraged by the TME. For instance, the ability of the immune cell compartment to operate can be affected by tumor cell absorption of lipids secreted by stromal cells.^78^

Clinical and experimental studies have indicated that cholesterol plays an essential role in cancer development by activating oncogenic pathways such as mTORC1 signaling and Hedgehog,^79^^,^^80^ as well as encouraging epithelial to mesenchymal transition invasion and transition.^81^ Cholesterol, as demonstrated in breast cancer models, may also facilitate spread by reducing immune cell activity or increasing the ability of metastatic cells to survive ferroptosis,^82^ a type of iron-dependent programmed cell death characterized by the accumulation of lipid peroxides from membranes.

The most common types of lipids include phospholipids, sphingolipids, fatty acids (FAs), sterols, and triglycerides (fats and oils). Phospholipids and sphingolipids are the primary components of lipid bilayers in cell membranes, but when paired with other lipid signaling molecules, they can act as signaling molecules.^83^ The multifunctional enzyme FA synthase (FASN) in the cytoplasm converts acetyl-CoA to the 16-carbon SFA palmitic acid, which is used to produce FA. Malignant cells and cancer progression are usually associated with FASN overexpression and hyperactivity.^84^

Lipid metabolism is used by cancer cells as a means of overcoming various obstacles encountered during the metastatic cascade. During secondary outgrowth, lipid intake and storage provide both the energy needed to maintain movement and the building blocks required for membrane formation. Metastatic cells regulate their lipid metabolism, altering the composition of their lipid membranes and producing metabolic intermediates that enable them to withstand the oxidative conditions that arise during their dispersion and separation from the tumor matrix. Crucially, alterations in the lipid composition of the primary tumor and the premetastatic niche may increase the capacity of cancer cells to proliferate and elude immune monitoring.^83^

Intratumoral Tregs are immunomodulators that activate the transcription factors CD36 and PPAR-b, which increase fatty acid oxidation (FAO) and lipid absorption.^85^ To boost FASN-dependent lipid production and PD-1 (programmed cell death protein 1)-mediated inhibitory receptor signaling, these cells can up-regulate the SREBP transcriptional program.^86^ By increasing lipid droplet accumulation,^87^ FAO,^88^^,^^89^ and CD36 expression,^88^ TAMs increase lipid metabolism. Similarly, intra-tumor MDSCs overexpress FATP1/2 or CD36, which enables them to produce prostaglandin E2 and absorb and retain lipids. Their ability to suppress the immune system is gradually increasing, which encourages the formation of tumors.^90^^,^^91^

## Mechanisms of ferroptosis resistance in the digestive system

Ferroptosis plays a vital role in tumorigenesis, progression and chemotherapy resistance, particularly in digestive malignancies, where its resistance mechanism becomes a significant obstacle to treatment failure and tumor recurrence. A key finding of this study is that ferroptosis resistance constitutes an essential mechanism by which cancer cells survive exposure to cytotoxic therapeutic agents, effectively undermining treatment efficacy and contributing to both intrinsic and acquired drug resistance in cancers. The mechanisms of ferroptosis resistance and cancer drug resistance related to ferroptosis in the digestive system are summarized in Table 1.

**Table 1:** Mechanisms of ferroptosis resistance and cancer drug resistance related to ferroptosis in the digestive system.

Core Mechanism	Key Targets/Pathways	Cancer Types	Resistance Mechanism	Ref
GPX4 pathway regulation	GPX4↑ (Wnt/β-catenin)	GC	Direct GPX4 up-regulation	99
	NeuroD1→GPX4↑	HCC	Transcriptional activation of GPX4	105
	CYP2J2/EETs→PPARγ→GPX4↑	PDAC	PPARγ-dependent GPX4 induction	113
	E-cadherin/β-catenin→GPX4↑	CRC	*F. nucleatum*-mediated GPX4 upregulation	129
Lipid peroxidation suppression Lipid peroxidation suppression	miR-522→ALOX15↓ (CAF-derived)	GC	Inhibits lipid-ROS accumulation	102
	SLC25A42→fatty acid↑	GC	Reduces free fatty acids and ROS	103
	LDs→FSP1↑	GC	Inhibits ferroptosis	104
	TIGAR→ROS/AMPK→SCD1↑	CRC	Blocks lipid peroxidation via SCD1	127
	Nodal→SCD1↑	CRC	Reduces sensitivity to RSL3-induced ferroptosis	126
	Zinc→Lactate→SREBP1/SCD1↑	ESCC	Metabolic reprogramming inhibits ferroptosis	94
	B7H3→SREBP2→Cholesterol metabolism↓	CRC	Inhibits cholesterol-driven ferroptosis	125
Iron metabolism dysregulation	SLC12A5→ER stress & cystine transport↑	HCC	Alters iron/cystine homeostasis	109
	FAM98A→xCT↑	CRC	Enhances cystine uptake via stress granules	130
	Lipocalin 2→Iron↓, GPX4/xCT↑	CRC	Reduces intracellular iron levels	128
Autophagy/Lysosomal dysfunction	CircHIPK3→miR-508-3p→Bcl-2/beclin1/SLC7A11	GC	Blocks autophagy-dependent ferroptosis	100
	LOXL3→DHODH stabilization↑	HCC	Inhibits mitochondrial ferroptosis	111
	CISD2→Autophagy regulation	HCC	Promotes sorafenib resistance	131
Tumor microenvironment crosstalk	CAF-secreted cysteine (TGF-β/SMAD3/ATF4)	PDAC	Enhances antioxidant capacity	116
	Stellate cell→HGF secretion	PDAC	Paracrine HGF increases antioxidant defense	114
	DACT3-AS1↓ in CAF exosomes (miR-181a-5p/SIRT1)	GC	Promotes malignant transformation	101
Oncogenic signaling activation Oncogenic signaling activation Oncogenic signaling activation	USP8→β-catenin stabilization↑	HCC	Confers resistance via the Wnt pathway	106
	YY1/p53 pathway↑	GC	Mediates apatinib resistance	132
	p53/SLC7A11 axis↓	ESCC	Confers paclitaxel resistance by inhibiting ferroptosis	96
	MiRNA axis → CisR-exo↑	ESCC	Confers cisplatin resistance	95
	CBX3/NRF2/GPX2 axis↑	CRC	Suppresses ferroptosis	121
	p52-ZER6/DAZAP1/SLC7A11 axis↑	CRC	Reduces lipid ROS	122
	PRMT5↑	CRC	by histone modifications	123
	YAP/TAZ→ATF4↑	HCC	Prevents ferroptosis in sorafenib resistance	133
	Galectin-1→MET/AXL↑	HCC	Enhances sorafenib resistance	134
RNA/Epigenetic regulation	ETS1/miR-23a-3p→ACSL4↓	HCC	Attenuates ferroptosis	108
	HBV→SRSF2→PCLAF splicing↑	HCC	Reduces ferroptosis via abnormal splicing	135
	LncRNA MACC1-AS1↑	PDAC	Suppresses ferroptosis to induce gemcitabine resistance	136
	SNHG4→PTEN↓	CRC	Causes PTEN instability to block ferroptosis	137
Metabolic reprogramming	FAM60A-PPAR axis↑	PDAC	Nutrient-deficient microenvironment inhibits ferroptosis	115
	CPT1B→KEAP1/NRF2→Redox homeostasis↑	PDAC	Maintains redox balance to inhibit ferroptosis	138
	CAF exosomal miRNAs→ACSL4↓	PDAC	Suppress ferroptosis to induce gemcitabine resistance	139
Survival pathways Survival pathways	GABARAPL1↓	HCC	Confers resistance in cancer stem-like cells	110
	BUB1↑	PDAC	Inhibits ferroptosis to promote gemcitabine resistance	140
	RBCK1→MFN2 degradation↑	PDAC	Disrupts mitochondrial fusion	112
Drug efflux	ABCC5→SLC7A11 inhibition↑	HCC	Blocks SLC7A11-induced ferroptosis	141
	CYP1B1→ACSL4 degradation↑	CRC	Induces anti-PD-1 resistance	142
Stress response Stress response	Tmem39b↑	HCC	Promotes sorafenib resistance by inhibiting ferroptosis	143
	DUSP4↑	HCC	Suppresses ferroptosis and enhances sorafenib resistance	144
	METTL16↑	HCC	Prevents ferroptosis to promote tumorigenesis	107
	ABHD12↑	HCC	Prevents ferroptosis to promote tumorigenesis	145
Exosomal communication	Exosomal circUPF2↑	HCC	Redeploys ferroptosis sensitivity	146

### Esophageal squamous cell carcinoma

One common type of cancer in the digestive system is esophageal squamous cell carcinoma (ESCC).^92^ The death rate for ESCC patients is still high due to the absence of distinctive early signs and viable therapy targets.^93^ As a result, developing novel therapeutic targets and new treatments has become an urgent issue. According to a recent study, ferroptosis resistance is conferred by ZD-enhanced ESCC glycolysis and lactate generation, which blocks p-AMPK and up-regulates SREBP1 and SCD1 in esophageal squamous cell carcinoma.^94^ Cisplatin-resistant cells release CisR-exo, which is enriched with miR-130a-3p. Consequently, the delivery of miR-130a-3p via CisR-exo confers cisplatin resistance both *in vitro* and in xenograft models, highlighting the role of the exosome-miRNA-m^6^A axis in chemoresistance.^95^ ANP32E overexpression in ESCC promotes cancer progression and paclitaxel resistance by inhibiting ferroptosis through down-regulation of the p53/SLC7A11 axis. Combinatorial treatment with paclitaxel and the ferroptosis inducer erastin potently inhibits tumor growth *in vivo*, underscoring the therapeutic potential of targeting this axis.^96^

### Gastric cancer

Gastric cancer (GC) is the fifth most prevalent malignancy and the fourth major cause of mortality from malignant neoplasms globally,^97^ and advanced unresectable or recurrent advanced GC has a dismal prognosis,^98^ necessitating further treatment research. In gastric cancer, targeting GPX4 can confer resistance to ferroptosis by inhibiting the abnormal stimulation of Wnt/beta-catenin signaling.^99^ CircHIPK3 suppressed autophagy-dependent ferroptosis via the miR-508-3p/Bcl-2/beclin1/SLC7A11 axis, making GC/DDP cells more resistant to cisplatin.^100^ Through its targeting of the miR-181a-5p/sirtuin 1(SIRT1) axis, DACT3-AS1 inhibited cell proliferation, migration, and invasion. In gastric cancer, the loss of cancer-associated fibroblast-derived exosomal DACT3-AS1 stimulated malignant transformation and ferroptosis-mediated oxaliplatin resistance.^101^ By stimulating the USP7/hnRNPA1 axis, cisplatin and paclitaxel increased the production of miR-522 from CAFs. This, in turn, reduced ALOX15 and the accumulation of lipid-ROS in cancer cells, thereby preventing ferroptosis and ultimately leading to acquired drug resistance.^102^ Multiple studies have focused on lipid metabolic adaptations as central to ferroptosis evasion in GC. SLC25A42 promotes fatty acid oxidation via CPT2 up-regulation and acetylation, enhancing mitochondrial respiration while reducing free fatty acids and ROS, thereby conferring ferroptosis resistance and supporting tumor growth.^103^ Similarly, during peritoneal metastasis, GC cells accumulate lipid droplets (LDs) via GPD1/GPD1L-mediated triglyceride synthesis. LD formation sequesters polyunsaturated fatty acids away from peroxidation and stabilizes ferroptosis suppressor protein 1 (FSP1) by inhibiting its ubiquitination. FSP1 up-regulation potently inhibits ferroptosis, facilitating anoikis resistance and metastasis.^104^

### Hepatocellular carcinoma

Hepatocellular carcinoma (HCC) poses a significant threat to human health. Therefore, it is crucial to investigate the causes of hepatocellular carcinoma thoroughly. Numerous studies conducted in recent years have discovered signaling pathways in hepatocellular carcinoma that can result in resistance to ferroptosis.^105^^,^^106^ Huang et al identified a novel function of NeuroD1 in the transcriptional regulation of GPX4, showing that it directly binds to the GPX4 promoter and triggers its transcriptional activity, ultimately causing ferroptosis resistance in cancer cells. By focusing on the NeuroD1/GPX4 axis, the study clarified the fundamental mechanism of ferroptosis in cancer cells and provided an achievable new strategy for future treatments.^105^ High METTL16 expression conferred ferroptosis resistance, promoted tumor growth, and was associated with a poor prognosis.^107^ Tang et al found that USP8 imparts ferroptosis resistance and favorably influences the development of hepatocellular carcinoma based on Wnt/beta-catenin signaling.^106^ This research raised the possibility of adopting a USP8 inhibitor as a therapeutic strategy to treat HCC. In 2022, Lu et al showed that miR-23a-3p overexpression could attenuate ferroptosis, and it was possible to stimulate ferroptosis via ETS1/miR-23a-3p/ACSL4.^108^

Numerous studies have been conducted to gain a deeper understanding of ferroptosis resistance in areas beyond signaling pathways. According to Tong et al, elevated levels of SLC12A5 in HCC prevented ferroptosis, stimulated cancer development, and were associated with a poor prognosis.^109^ These results provide insight into ferroptosis in HCC and show that SLC12A5 is a therapeutic target. The loss of ferroptosis-related stemness genes, such as GABARAPL1, conferred ferroptosis resistance to cancer stem-like cells.^110^ A new study discovered that lysyl oxidase-like 3 inhibits mitochondrial ferroptosis by stabilizing dihydroorotate dehydrogenase.^111^ Hence, they represent potentially effective therapeutic targets for reducing sorafenib resistance in patients with HCC.

### Pancreatic ductal adenocarcinoma

As one of the most severe cancers of the digestive tract, pancreatic ductal adenocarcinoma (PDAC) has a poor prognosis and a high recurrence rate. Pancreatic ductal adenocarcinoma is particularly susceptible to ferroptosis. However, ferroptosis resistance has been observed in pancreatic ductal adenocarcinoma in numerous studies. According to Su et al, ferroptosis resistance in pancreatic cancer was conferred by the E3 ubiquitin ligase RBCK1, which facilitates MFN2 degradation. This suggest that targeting the RBCK1-MFN2 axis could be an affordable therapeutic approach for PDAC patients.^112^ PDAC tissues have high levels of CYP2J2/EET expression. According to a different study, ferroptosis was inhibited by epoxyeicosatrienoic acids (EETs) through the up-regulation of GPX4 in a PPARγ-dependent manner. This mechanism was thought to contribute to the resistance of PDAC to ferroptosis.^113^ Furthermore, a study found that pancreatic cancer cells might mediate ferroptosis resistance in pancreatic cancer and activate pancreatic stellate cells, encouraging their production of HGF and increasing their antioxidant capacity. In light of the fibrotic microenvironment associated with pancreatic cancer, it may provide an achievable therapeutic alternative.^114^ According to Pan et al, FAM60A inhibited ferroptosis through the GPX4 and PPAR signaling pathways, thereby increasing the likelihood of cancer development.^115^ From another perspective, Zhu et al discovered that CAFs secrete cysteine, which is taken up by pancreatic cancer cells and metabolized into glutathione, thereby enhancing cancer resistance to ferroptosis and cisplatin chemotherapy, playing a crucial role in PDAC metabolism.^116^ Mechanistically, they demonstrated that pancreatic cancer cell-derived TGF-β activates SMAD3 signaling in CAFs. This activation induces the expression of the transcription factor ATF4, which directly binds to the promoters of key transsulfuration pathway enzymes, cystathionine β-synthase (CBS) and cystathionase (CTH), up-regulating their expression. Consequently, increased CBS/CTH-dependent cysteine synthesis in CAFs drives cysteine secretion, primarily mediated by transporters like SLC7A11. Targeting this TGF-β/SMAD3/ATF4 axis or directly inhibiting CBS effectively suppressed cysteine secretion, reversed ferroptosis resistance, and sensitized PDAC to chemotherapy *in vitro* and *in vivo*, offering novel therapeutic strategies for treating pancreatic cancer.

Currently, the most widely used chemotherapy medication for pancreatic cancer is gemcitabine. Unfortunately, the response rate to gemcitabine for pancreatic cancer is less than 20 % overall, and 80 % of patients die within a year of recurrence.^117^ The discovery of several pathways that lead to gemcitabine resistance is significant for the development of treatments aimed at overcoming chemoresistance.

### Colorectal cancer

Globally, colorectal cancer (CRC) is the fourth most prevalent cause of cancer-related mortality and the third most frequently diagnosed cancer with almost 2 million new cases diagnosed annually.^3^^,^^118^ A complicated interaction between hereditary and environmental variables, such as age, obesity, eating habits, and a sedentary lifestyle, leads to the development of colorectal cancer.^119^ About 40% of patients with localized disease develop recurrence after previous therapy, and 20% of cases are diagnosed as metastatic colorectal cancer due to its subtle starting.^120^ In exploring ferroptosis resistance mechanisms in CRC, recent studies have revealed several pivotal pathways and molecular interactions that contribute to CRC progression and chemoresistance. One study highlighted the CBX3/NRF2/GPX2 axis, showing that CBX3 upregulates NRF2 by inhibiting CUL3 transcription, which in turn enhances GPX2 expression, thereby suppressing ferroptosis and promoting multidrug resistance in CRC cells.^121^ Another study identified the p52-ZER6/DAZAP1/SLC7A11 axis, where p52-ZER6 enhances DAZAP1 transcription, leading to increased SLC7A11 mRNA stability and expression, thereby reducing lipid ROS accumulation and enhancing ferroptosis resistance.^122^ Additionally, research on PRMT5 revealed its role in promoting ferroptosis resistance through the ALKBH5/SLC7A11 axis via histone modifications, with PRMT5 K240lac being crucial for this interaction.^123^ The microbial metabolite trans-3-indoleacrylic acid (IDA) from P.anaerobius inhibited ferroptosis, promoting colorectal cancer.^124^ Jin et al discovered that B7H3 may regulate ferroptosis resistance in CRC by controlling SREBP2-mediated cholesterol metabolism, thereby promoting ferroptosis resistance.^125^ Stearoyl-coenzyme SCD1 suppression reduced the resistance of Nodal-overexpressing cells to RSL3-induced ferroptosis, which in consequence improved colorectal cancer metastasis and survival.^126^ Moreover, TIGAR induced ferroptosis resistance in CRC cells through the ROS/AMPK/SCD1 signaling pathway.^127^ Lipocalin 2 prevented ferroptosis by lowering intracellular iron levels and increasing the production of glutathione peroxidase 4 and xCT.^128^

## Mechanisms of ferroptosis reversing cancer drug resistance

Even with significant therapy improvements, cancer drug resistance remains one of the greatest obstacles in cancer treatment today. Overcoming drug resistance is the focus of considerable clinical research. Additionally, ferroptosis has recently been shown to reverse drug resistance in cancer treatments. The mechanisms of reversing cancer drug resistance related to ferroptosis in the digestive system are outlined in Table 2.

**Table 2:** Mechanisms of reversing cancer drug resistance related to ferroptosis in the digestive system.

Intervention Strategy	Cancer Type	Chemotherapeutic Drug	Key Target	Reversal Mechanism	Ref
Gene silencing Gene silencing	ESCC	Cisplatin	ALDH5A1 knockdown	Activates ferroptosis signaling	149
	ESCC	Sorafenib	Apoc1 inhibition	Promotes sorafenib-induced ferroptosis via GPX4	151
	GC	Capecitabine	ARF6 knockdown	Promotes erastin-induced ferroptosis	157
	GC	Sorafenib	SIRT6 knockdown	Targets SIRT6/Keap1/Nrf2/GPX4 axis	158
	HCC	Sorafenib	lncRNA HCG18 silencing	Inhibits GPX4 by adsorbing miR-450b-5p	167
	PDAC	Gemcitabine	SLC38A5 knockdown	Induces ferroptosis in gemcitabine-resistant cells	174
	PDAC	Gemcitabine	cTRIP12	Reduces FTH and PD-L1 expression	178
	CRC	Oxaliplatin	CDK1 inhibition	Restores ACSL4-mediated ferroptosis	182
Small molecule inducers Small molecule inducers Small molecule inducers	ESCC	Paclitaxel	Verteporfin	Induces ferroptosis and reverses paclitaxel resistance	150
	GC	Cisplatin	ATF3 elevation	Induces ferroptosis via Nrf2/Keap1/xCT suppression	156
	GC	Cisplatin	PFKFB3	Enhances cystine uptake and GSH synthesis	154
	GC	Cisplatin	FAM120A deficiency	Promotes ferroptosis to reduce cisplatin resistance	155
	HCC	Sorafenib	Glycyrrhizic acid	Triggers ferroptosis via mTOR signaling	169
	HCC	Sorafenib	Withaferin A	Modulates Keap1/Nrf2-associated EMT and ferroptosis	170
	PDAC	Gemcitabine	SIK1	Activates ferroptosis	173
	CRC	Erastin	GCH1/BH4 axis blockade	Activates ferroptosis by blocking ferritinophagy	185
RNA-based therapy	HCC	Sorafenib	miR-654-5p (engineered sEVs)	Targets HSPB1 to induce ferroptosis	171
	HCC	Lenvatinib	HAND2-AS1→TLR4/NOX2/DUOX2	Promotes ferroptosis by competing with miR-219a-1-3p	172
RNA-based therapy	HCC	Sorafenib	lncRNA MALAT1	Promotes the expression of SLC7A11 via ELAVL1	164
	HCC	Sorafenib	HNF4A-AS1	Promotes ferroptosis	166
Combination therapy	CRC	5-Fluorouracil	Jianpi Jiedu decoction	Suppresses xCT/GSH/GPX4 axis	179
	CRC	Cetuximab	3-Bromopyruvate	Induces autophagy-dependent ferroptosis	183
Novel compounds Novel compounds	PDAC	Cisplatin	Ligustrazine-derived Pt (IV)	Synergizes ferroptosis and apoptosis	177
	CRC	Oxaliplatin	Novel Pt (IV) complexes	Induces ferroptosis and apoptosis	180
	CRC	Oxaliplatin	RBMS1/PRNP axis inhibition	Activates ferroptosis	181
	CRC	Oxaliplatin	BRAF ± EGFR inhibitors	Up-regulates GPX4 expression	184
	PDAC	Gemcitabine	ACADM knockdown	Modulates fatty acid metabolism and ferroptosis	175
	PDAC	Gemcitabine	ARF6 knockdown + RSL3	Amplifies RSL3-induced ferroptosis	176

### Esophageal squamous cell carcinoma

The majority of ESCC patients are diagnosed at an advanced stage and are not eligible for resection since they are primarily asymptomatic, which leads to a poor prognosis and increased mortality.^147^ For ESCC patients who are unable to tolerate radiotherapy or surgical resection, chemotherapy has been found to be a reasonably curative treatment option. For the treatment of ESCC, 5-fluorouracil (5-FU), cisplatin, and adriamycin or 5-FU, cisplatin, and paclitaxel are used as integrated chemotherapy. However, due to primary or acquired drug resistance, several patients receiving chemotherapy were unable to achieve a better prognosis.^148^ Thus, it is crucial to identify robust biosignatures for ESCC to overcome chemoresistance and improve therapeutic outcomes. Experiments have shown that ALDH5A1 can act as an oncogene in ESCC chemoresistance. Ferroptosis signaling pathways can be activated by silencing ALDH5A1, which may decrease cisplatin resistance in ESCC.^149^ Wang et al found that treatment with verteporfin (VP) resulted in notable ferroptosis events, which inhibited the survival of ESCC cells and reversed their resistance to paclitaxel by inducing ferroptosis.^150^ Sorafenib resistance in EC cells can be eliminated by reducing Apoc1, which promotes sorafenib-induced ferroptosis via GPX4.^151^

### Gastric cancer

Gastric cancer is the fifth most prevalent malignancy and the fourth major cause of mortality from malignant neoplasms globally.^97^ Early diagnosis and treatment, including medication and surgery, improve the prognosis for GC patients. However, most stomach cancer patients are diagnosed at an advanced stage.^152^ The main obstacle in GC therapy is intrinsic or acquired chemoresistance^153^; therefore, exploring new chemoresistance treatments is crucial. Geng et al reported that YY1 overexpression prevents immune cell infiltration in GC cancers. Furthermore, GC cell ferroptosis was inhibited by YY1 overexpression, and apatinib resistance was mediated through the p53 pathway.^132^ Direct modification of key ferroptosis regulators is another mechanism of resistance. PFKFB3 desensitizes GC cells to cisplatin by directly dephosphorylating SLC7A11/xCT at serine 26 via its phosphatase domain. This activates SLC7A11, enhancing cystine uptake and GSH synthesis, which blunts cisplatin-induced ferroptosis.^154^ In contrast, novel studies found that by enhancing ferroptosis through Nrf2/Keap1/xCT suppression, elevated ATF3-sensitized cisplatin-resistant GC cells to cisplatin, and FAM120A deficiency can reduce cisplatin resistance by promoting ferroptosis.^155^^,^^156^ Thus, ferroptosis has emerged as a promising approach to reverse cancer drug resistance in gastric cancer. ARF6 silencing increased ferroptosis induced by erastin and markedly decreased resistance to capecitabine.^157^ By targeting the SIRT6/Keap1/Nrf2/GPX4 signaling pathway, SIRT6 knockdown overcame sorafenib resistance and promoted ferroptosis.^158^ A recent study has shown that targeting SOX13 can inhibit the assembly of respiratory chain supercomplexes and overcome ferroptosis resistance in gastric cancer. The research indicated that SOX13 enhances protein remodeling of electron transport chain complexes by directly transactivating SCAF1, leading to increased supercomplex assembly, enhanced mitochondrial respiration, and increased chemoresistance.^159^ Another study has demonstrated that POLQ positively regulates stem cell-like characteristics and ferroptosis resistance in gastric cancer cells. POLQ inhibition was found to down-regulate DHODH expression, which is a critical factor in ferroptosis resistance.^160^ All of these studies provide potential therapeutic targets for overcoming ferroptosis resistance in gastric cancer.

### Hepatocellular carcinoma

Since HCC is an inherently drug-resistant cancer, most HCC patients are not susceptible to chemotherapeutic medications. They are more likely to become MDR while receiving chemotherapy, which lowers survival and improves prognosis.^161^ The most popular option for targeted therapy for hepatocellular carcinoma is sorafenib. However, sorafenib resistance during HCC chemotherapy is a common and serious problem that has a significant impact on medical treatment. Research has revealed that the expression of Tmem39b, CISD2, miR-23a-3p, dual-specific phosphatase 4 (DUSP4), and exosomal circUPF2 can increase resistance to sorafenib.^108^^,^^131^^,^^143^^,^^144^^,^^146^ Furthermore, NCOA5, CircTTC13, ABHD12, galectin-1-mediated, HBV, ABCC5, and YAP/TAZ prevent ferroptosis, which increases resistance to sorafenib in hepatocellular carcinoma.^133^, ^134^, ^135^^,^^141^^,^^145^^,^^162^^,^^163^ It has been demonstrated that long non-coding RNAs and RNA-binding proteins play crucial roles in regulating ferroptosis. For instance, the lncRNA MALAT1 is up-regulated in sorafenib-resistant HCC cells through m5C methylation by NSUN2 and ALYREF. MALAT1 inhibits sorafenib-induced ferroptosis by promoting the expression of SLC7A11 via ELAVL1, leading to increased intracellular GSH levels and reduced lipid reactive oxygen species.^164^ On the other hand, HNF4A-AS1, a lipid metabolism-related lncRNA, contributes to sorafenib resistance by decreasing PUFA content and promoting resistance to ferroptosis when its expression is down-regulated.^165^ Additionally, the RNA-binding protein MVP has been identified as an oncogenic RBP that suppresses ferroptosis by binding to LCN2 mRNA and maintaining its stability. This interaction is facilitated by PGAM5-mediated dephosphorylation of MVP.^166^ These findings collectively suggest that targeting these lncRNAs and RBPs could be a promising strategy to enhance the efficacy of sorafenib and overcome resistance in HCC. Fortunately, studies have shown that glycyrrhizic acid reduces sorafenib resistance by triggering ferroptosis through mTOR signaling, while Withaferin A may reduce metastatic potential and sorafenib resistance by modulating Keap1/Nrf2-associated EMT and ferroptosis. Silencing HCG18 inhibited GPX4 by binding to miR-450b-5p, increased GPX4-inhibited ferroptosis, and prevented sorafenib resistance in HCC.^167^ Besides, HCG18 can inhibit ferroptosis by regulating the expression of RRM2, thereby promoting HCC proliferation; thus, it holds promise as a potential target for ferroptosis-dependent therapy.^168^ Meanwhile, m654-sEV effectively distributed miR-654-5p to HCC cells, targeting HSPB1 and increasing ferroptosis to reduce sorafenib resistance,^167^^,^^169^, ^170^, ^171^ miR-654-5p reduces HSPB1 protein levels and thereby increases sorafenib-induced ferroptosis.^15^ Besides, Song et al found that HAND2-AS1 increases the expression of ferroptosis-related genes and prevents lenvatinib resistance by enhancing TLR4/NOX2/DUOX2 by competing with endogenous miR-219a-1-3p in HCC cells.^172^

### Pancreatic ductal adenocarcinoma

Gemcitabine is used to treat advanced metastatic patients; however, its results are not satisfactory, and this is primarily attributed to the development of resistance to treatment. Gemcitabine resistance in pancreatic cancer cells was induced by the inhibition of ferroptosis by cancer-associated fibroblasts that secrets exosome-derived ACSL4-targeting miRNAs and lncRNA MACC1-AS1.^136^^,^^139^ Moreover, Abudureyimu Tuerhong et al discovered that CPT1B could cause gemcitabine resistance in pancreatic cancer via the KEAP1/NRF2 axis,^138^ and targeting SIK1 could trigger lethal ferroptosis and render PDAC cells vulnerable to gemcitabine.^173^ However, researchers have identified novel targets to overcome resistance to gemcitabine. Gemcitabine-resistant patients exhibit a higher level of overexpression of the glutamine transporter SLC38A5 than the gemcitabine-sensitive patients. Gemcitabine-resistant PDAC cell migration and proliferation were reduced by the loss of SLC38A5, which induces ferroptosis in gemcitabine-resistant pancreatic cancer cells.^174^ By producing ferroptosis, ACADM knockdown amplified the cytotoxic effects initiated by FA, while ARF6 knockdown amplified the ferroptosis induced by RSL3, which significantly mitigated gemcitabine resistance.^175^^,^^176^ Additionally, Wang et al demonstrated how ferroptosis enables the intervention of multitarget platinum (IV) prodrugs in cisplatin resistance in pancreatic cancer.^177^ A study found that cTRIP12 inhibition induced ferroptosis in PDAC cells by reducing FTH and PD-L1 expression and synergistically increased the efficacy of immunotherapy.^178^ These discoveries are significant for the development of treatments aimed at overcoming chemoresistance in pancreatic cancer.

### Colorectal cancer

In CRC cells, SNHG4 conferred oxaliplatin resistance by blocking ferroptosis through PTEN instability.^137^ Through the E-cadherin/β-catenin/TCF4 pathway, *F. nucleatum* mechanically increased GPX4 expression, which helps inhibit L-OHP-induced ferroptosis and eventually results in oxaliplatin resistance.^129^ Furthermore, increased FAM98A expression prevented ferroptosis in CRC cells by triggering xCT translation in stress granules, ultimately enhancing resistance to 5-fluorouracil.^130^ Recent research has indicated that CYP1B1 generates anti-PD-1 resistance and enhances CRC cell resistance to ferroptosis. Individuals with high CYP1B1 expression had a disappointing prognosis.^142^ Fortunately, Ou et al found that combining Jianpi Jiedu decoction with 5-FU treatment induced cell death and reversed 5-FU resistance in colorectal cancer.^179^ Oxaliplatin is a widely used chemotherapy drug for patients with colorectal cancer. It was discovered that utilizing novel platinum (IV) complexes and inhibiting the RBMS1/PRNP axis overcame oxaliplatin resistance in colorectal cancer by activating ferroptosis.^180^^,^^181^ Meanwhile, CDK1 was required for oxaliplatin resistance in CRC via the inhibition of ACSL4-mediated ferroptosis. Knocking down CDK1 restores the susceptibility of CRC cells to oxaliplatin treatment.^182^ The combination of 3-BP with cetuximab increased the cytotoxic effect on cetuximab-resistant human CRC cells and promoted ferroptosis, which eventually overcame cetuximab resistance in human colorectal cancer cells.^183^ Through genome-wide CRISPR-Cas9 screening, researchers discovered that targeting GPX4 can enhance the efficacy of BRAF ± EGFR inhibitors. The study reveals that BRAF ± EGFR inhibitors up-regulate GPX4 expression, which counteracts therapy-induced ferroptosis. Furthermore, it identifies a PLK1–CBX8–GPX4 signaling axis where PLK1 activation leads to CBX8 phosphorylation, promoting GPX4 transcription. The combination of PLK1 inhibitors with BRAF ± EGFR inhibitors can trigger ferroptosis and overcome resistance in BRAFV600E CRC.^184^ It was found that GCH1/BH4 metabolism inhibited the ferroptosis effect of erastin induction in colorectal cancer by blocking NCOA4-mediated ferritinophagy, offering a possible target for anticancer activity.^185^

## Conclusion and perspective

In recent years, ferroptosis has emerged as a significant area of research in cancer treatment. Ferroptosis is a type of programmed cell death caused by iron-dependent phospholipid peroxidation, which can be used to reverse drug resistance in cancer treatment. In this review, we provide a summary of the latest developments in the mechanisms underlying ferroptosis and drug resistance, and we identify ferroptosis resistance as the key mechanism of resistance to tumor treatment. In addition, we provide a detailed summary of the mechanism of ferroptosis resistance and how cancer resistance can be reversed by targeting ferroptosis in cancers of the digestive system.

Furthermore, we believe that the application of ferroptosis features in conjunction with additional therapies, including immunotherapy, targeted therapy, radiation, and chemotherapy, leads to a comprehensive treatment that improves therapeutic outcomes.^186^ According to a recent report, anti-PD-L immune checkpoint inhibition can promote ferroptosis responses in cancer cells by suppressing the expression of SLC7A11 in cancer cells owing to the release of IFN-γby CD8^+^T cells.^187^

Current treatments have limited effects on the ferroptosis of DT cells, indicating the need for further research and development in the field of ferroptosis inducers and targets. Ferroptosis research may be crucial in the therapeutic situation to increase the effectiveness of cancer treatment. For instance, certain methods can cause ferroptosis in cancer cells to increase the effectiveness of chemotherapy or radiation therapy and lower the risk of cancer spread and recurrence. Comprehensive research into the ferroptosis process can also help in the developement of new anticancer medications and expand the range of available treatments for patients with refractory cancers. In the future, tailored treatment plans based on individual differences might be created with a deeper comprehension of the expression patterns of ferroptosis-related genes and proteins. Ferroptosis inducers or combination treatments most suitable for a particular patient can be selected using a precision medicine approach. However, significant challenges remain in translating ferroptosis-based therapies into clinical practice. First, the development of reliable biomarkers to predict ferroptosis sensitivity is complicated by metabolic heterogeneity within tumors and dynamic changes in the tumor microenvironment. Second, the systemic toxicity of ferroptosis inducers necessitates innovative delivery strategies, such as nanoparticle encapsulation or cancer-targeted activation, given that iron overload can cause hepatorenal damage and that GPX4 inhibition poses risks to organ toxicity. Additionally, balancing therapeutic efficacy with potential oxidative damage to normal tissues necessitates careful dose optimization and the use of combinatorial regimens that incorporate antioxidants for the protection of non-malignant cells.

Although the basic features and some regulatory mechanisms of ferroptosis have been elucidated, further research is necessary to gain a deeper understanding of the detailed molecular mechanisms by which ferroptosis influences treatment resistance in digestive system cancers. It is essential to note, however, that despite encouraging research on ferroptosis as a potential approach to reversing cancer drug resistance, many of the findings in this area are still in the laboratory stage and have not yet been translated into practical clinical applications. Therefore, it is critical to design rational clinical trials to validate the efficacy and safety of ferroptosis inducers.

## CRediT authorship contribution statement

**Wenjie Chen:** Writing – review & editing, Writing – original draft, Project administration, Conceptualization. **Liang Han:** Writing – review & editing, Writing – original draft. **Jizhou Wang:** Writing – review & editing, Writing – original draft. **Linjiang Song:** Writing – review & editing, Funding acquisition, Conceptualization.

## Funding

The work is supported by the National Natural Science Foundation of China (No. 82305011).

## Conflict of interests

The authors declare no competing interests.
